# Systemic sclerosis in sub-Saharan Africa: a systematic review

**DOI:** 10.11604/pamj.2020.37.176.22557

**Published:** 2020-10-22

**Authors:** Julian Nicolas Erzer, Veronika Katharina Jaeger, Mohammed Tikly, Ulrich Andreas Walker

**Affiliations:** 1 Department of Rheumatology, University Hospital Basel, Basel, Switzerland,; 2 Institute of Epidemiology and Social Medicine, University of Münster, Münster, Germany,; 3 Division of Rheumatology, Chris Hani Baragwanath Academic Hospital, University of the Witwatersrand, Johannesburg, South Africa

**Keywords:** Systemic sclerosis, sub-Saharan Africa, connective tissue disease, systematic review

## Abstract

Systematic studies on connective tissue disorders are scarce in sub-Saharan Africa. Our aim was to analyse the published clinical data on systemic sclerosis (SSc) in sub-Saharan Africa. A systematic review was carried out in accordance with the PRISMA guidelines. We screened the Embase, PubMed and African Health Sciences databases for literature published until March 2018. Searches produced 1210 publications. After abstract and full-text screenings, 91 publications were analysed, and epidemiological information and clinical features extracted. Publications were mostly publications case reports (36%), cross-sectional studies (26%) and case series (23%) and came predominantly from South Africa (45%), Nigeria (15%) and Senegal (14%). A total of 1884 patients were reported, 66% of patients came from South Africa. The patients were between 4 and 77 years old; 83% of patients were female. Overall, 72% had diffuse SSc. Raynaud´s phenomenon was reported in 78% and skin ulcerations in 42% of patients. Focal skin hypopigmentation was common and telangiectasia not frequent. Interstitial lung involvement was reported in 50%, pulmonary hypertension in 30%, heart involvement in 28% of patients. Oesophageal reflux was observed in 70% and dysphagia in 37% of patients. Antinuclear antibodies were positive in 65% of patients. Anti-centromere autoantibodies (9.2%) and RNA polymerase 3 antibodies (7.1%) were rare and anti-fibrillarin most frequent (16.5%). SSc presentations in sub-Saharan Africa differ from those reported in Europe and America by a frequent diffuse skin involvement, focal skin hypopigmentation and a high prevalence of anti-fibrillarin autoantibodies.

## Introduction

With substantial advances in the prevention and treatability of infections, the non-communicable diseases have now emerged as an important contributor to the global disease burden and become a significant driver of health care costs in low and middle-income countries. Among the non-communicable diseases, some musculoskeletal disorders such as systemic sclerosis (SSc), are characterized by large unmet medical needs and growing proportions of global morbidity and mortality.

Systemic sclerosis (SSc) is a chronic, autoimmune disease with a multisystem vasculopathy and an immense increase of fibrous tissue in affected organs. Even in high-income societies, SSc has a poor prognosis, as about 55% of affected patients die as a direct consequence of their disease [1]. In America, African descendants are known to have a particularly high SSc incidence and also a more severe disease course than Caucasians [2]. Various studies have identified differences in genetic disease associations between Caucasians and African Americans. Two seminal observations were however made in Africa itself, namely the first description of the prominent visceral involvement in SSc, coining the term ‘progressive systemic sclerosis’ [3] and the association of SSc with silica dust exposure [4].

Although the epidemiology, clinical phenotype and evolution of SSc organ involvement are intensively researched in economically advantaged countries, further robust data of SSc presentations in sub-Saharan Africa are largely lacking, prompting us to undertake a systematic review.

## Methods

Information sources: we searched the Embase, PubMed, and African Health Sciences databases. Additionally, conference abstracts were retrieved by targeted searches of relevant websites, e.g. the African League against Rheumatism, the South African Rheumatism Arthritis Association, and the proceedings of the World Scleroderma Congresses. Reference lists of retrieved publications were also screened for publications not identified by the above search strategies. Search terms were defined (Table 1) including the corresponding Medical Subject Headings and Embase subject headings. A first search was carried out on August 31, 2017, and an update on March 29, 2018.

**Table 1:** overview of the search terms used to retrieve publications

Search terms
Scleroderma OR SSc OR systemic sclerosis OR CREST
**AND**
sub-Saharan Africa OR sub-Saharan Africa OR Central Africa OR Eastern Africa OR Western Africa OR Southern Africa OR Africa OR Djibouti OR Eritrea OR Ethiopia OR Somalia OR Somaliland OR Burundi OR Comoros OR Kenya OR Madagascar OR Malawi OR Mauritius OR Mozambique OR Rwanda OR Seychelles OR South Sudan OR Sudan OR Tanzania OR Zanzibar OR Uganda OR Zambia OR Zimbabwe OR Angola OR Cameroon OR Central African Republic OR Chad OR Congo OR Democratic Republic of the Congo OR DRC OR Zaire OR Equatorial Guinea OR Gabon OR Sao Tome OR Principe OR Botswana OR Lesotho OR Namibia OR South Africa OR Swaziland OR Benin OR Burkina Faso OR Cape Verde OR Cote d´Ivoire OR Ivory Coast OR Gambia OR Ghana OR Guinea OR Guinea Bissau OR Guinea-Bissau OR Liberia OR Mali OR Mauritania OR Niger OR Nigeria OR Senegal OR Sierra Leone OR Togo

Study selection and data collection: two reviewers (VKJ and JE) independently screened the titles and abstracts. Patients were eligible for the analysis, if they were diagnosed with SSc by the reporting physician and resided within sub-Saharan Africa. Disagreement between the reviewers was resolved by consensus. In a second step, inclusion into this review was based on the full text of the remaining publications (JE), cross-checked by another author (VKJ). Data was extracted by one author (JE) and checked by another author (VKJ). In case of disagreement, a consensus was sought by discussion between authors.

Data items: ‘SSc patients’ were defined as patients diagnosed with SSc by the reporting physician and included if they were residing in sub-Saharan Africa. Information extracted included study methodology, patient demographics, and disease presentations. The prevalences of symptoms and organ involvement were calculated as percentages based on the denominator of the number of patients with available information, rather than the total number of patients included.

## Current status of knowledge

**Figure 1:**
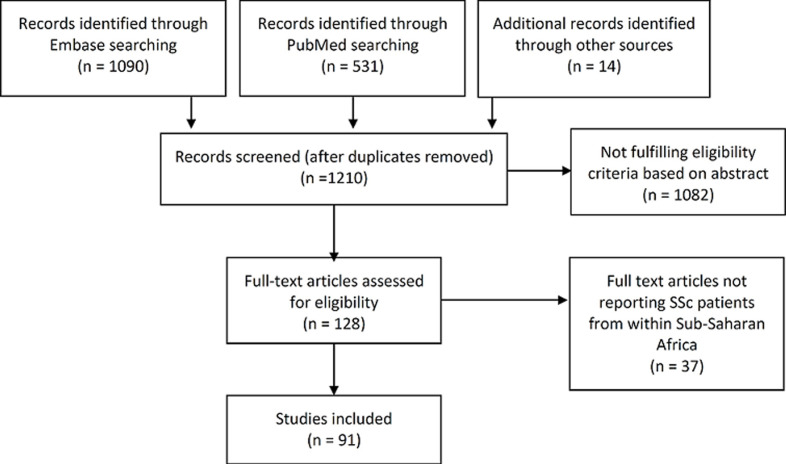
flow chart of paper collection and selection

Study selection: we retrieved 1090 publications from Embase, 531 from PubMed and 14 from other sources (Figure 1). After removing duplicate publications, 1210 publications were left for screening. The selection process finally included 91 papers in this review (Figure 1).

**Figure 2:**
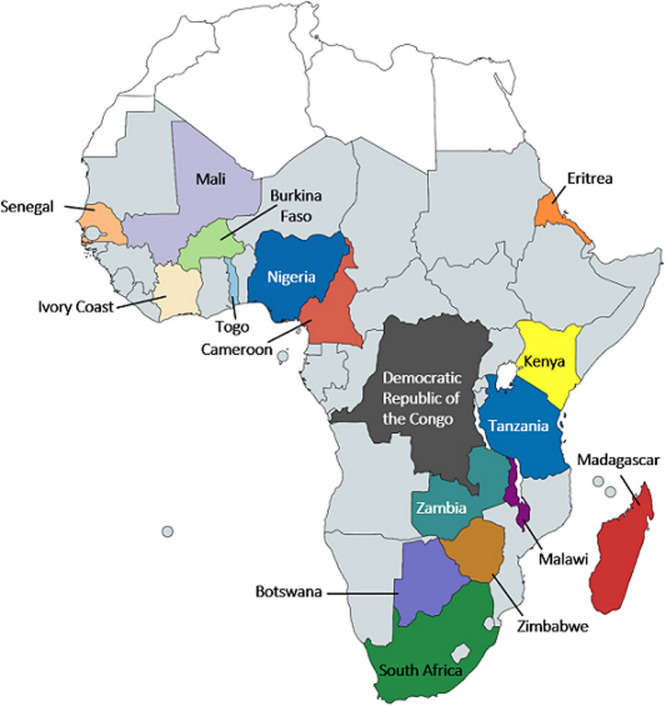
origin of publications and patients

Study characteristics: the 91 publications were published between the years 1945 [3] and 2018 [5], described a total of 1884 SSc patients and were carried out in 17 of the 48 sub-Sahara African countries (Figure 2), predominantly in South Africa (41 publications [45]), Nigeria (14 publications [15]) and Senegal (13 publications [14]).

About a third of the publications [36%] were case reports [3,6-37], 23% were case series [4,38-57], 24 publications [26%] were cross-sectional studies [5,58-81], 11 [12%] were case control studies [82-92]. There was one longitudinal cohort study [93] and one blinded cross-over interventional study [94]. Most studies were published in English (72 [79%]) and the remainder in French (19 [21%]).

Patient and disease characteristics: in almost a third of the studies (28 [31%]), patients fulfilled the 1980 ACR or 2013 ACR/EULAR classification criteria [95,96]; two [7% of publications] studies used the Medsger and Masi criteria [97]. In the remaining 60 publications [67%] the authors failed to describe the classification criteria. With respect to patient numbers, the figures were slighty different: 914 patients [48% of all 1884 patients] fulfilled the ACR or ACR/EULAR classification criteria for SSc, and 71 patients [4%] were classified according to the Medsger and Masi SSc criteria. For the remaining 899 patients [48%] there was no disease definition.

The majority of patients [66%] was reported in South Africa. The country of residence of all SSc patients is shown in Figure 2. The sex ratio was 5.4:1, 981 patients [83%] were female and 200 [17%] male, of the remaining 703 patients´ sex was not reported. The patient age ranged between 4 and 77 years. We estimate the mean patient age as approximately 40 years, but were unable to calculate this figure precisely as several studies reported only age ranges. The mean age at disease onset was about 36 years and the mean disease duration approximately 4 years (range 1 month to 19 years).

Most patients [72%] had diffuse cutaneous SSc (575 patients), 224 patients [28%] had limited SSc (Table 2), only two patients had SSc sine scleroderma [36,54]. One South African study analysed SSc subsets systematically and with a meaningful number (174 patients) and found a similar proportion of diffuse cutaneous SSc [74%] [5].

**Table 2:** clinical features of the 1884 SSc patients

Manifestation	Patients with available information N [% of all SSc patients]	Positive N [% of patients with available information]
Raynaud´s phenomenon	1085 [58]	851 [78]
Ulcers	727 [39]	307 [42]
Diffuse cutaneous SSc	801 [43]	575 [72]
Limited cutaneous SSc		224 [28]
SSc sine scleroderma		2 [0.2]
Reduced Forced Vital Capacity	184 [10]	183 [99]
Interstitial lung disease*	885 [47]	440 [50]
Pulmonary hypertension	576 [31]	173 [30]
Estimated by echocardiography		159 [92]
Diagnosed by right heart catheterisation		14 [8]
Heart involvement	524 [28]	144 [28]
Renal crisis	25 [1]	9 [36]
Dysphagia	417 [22]	153 [37]
Reflux	373 [20]	261 [70]
Oesophageal dysfunction	67 [4]	42 [63]
Diarrhoea	37 [2]	9 [24]
**Musculoskeletal involvement**		
‘Arthritis’	631 [33]	317 [50]
**Autoimmune overlap conditions**		
Systemic lupus erythematosus overlap	155 [8]	18 [12]
Rheumatoid arthritis overlap	159 [8]	5 [3]
Sjögren´s syndrome overlap	142 [8]	9 [6]
Dermatomyositis overlap	142 [8]	10 [7]
**Concomitant infections**		
Human Immunodeficiency Virus infection	153 [8]	9 [6]
Latent or active tuberculosis	45 [2]	22 [49]


* definition: radiographic evidence and/or reduced FVC in those with available information of at least one of the two items

Information on silica exposure was not available in 92% of study subjects, but was reported in 141 patients [8%] [4,45,48,49,91,92] which were mostly miners. Only 5 patients [0.3%] were reported to be free of silica exposure [14,21,50]. Two case control studies [91,92] suggested a substantially elevated SSc incidence in silica-exposed gold miners.

Raynaud´s phenomenon: RP was reported in 851 patients [78%]. In 11 patients there were symptoms that could be interpreted as RP, such as acrocyanosis in 3 patients [31,65] and digital ischaemia in 8 patients [17,52]. We had attributed these 11 patients to the 851 patients with RP. There was a wide geographical variation in RP prevalences. In a retrospective study conducted in Dakar, only 57% of 92 SSc patients had RP, [41] whereas in South Africa RP was reported in 92% of 153 patients, and in 94% of 34 patients [43,93].

Cutaneous manifestations: hypopigmented patchy skin lesions resembling vitiligo were reported in 7 of 13 SSc patients in a Togolese study [77]. A retrospective Senegalese study reported similar skin lesions in 70% of 92 patients [41]. Further reports of skin hypopigmentations with a ‘salt and pepper appearance’ were from Nigeria [8,46,54], Togo [51], and South Africa [36]. Papulous hypopigmentations were described as coexisting lichen sclerosus et atrophicus [18].

The extent of the skin fibrosis in terms of the mRSS [98] was only reported in two case reports [17,21] with scores of 18 and 23 and one case-control study in which the average mRSS was 24.7 [83]. The presence or absence of telangiectasia was documented in 321 patients [17%] of all patients]); in whom telangiectasia were present in 73 patients [23%]. In the publication with the most representative data, telangiectasia were present in only 19 of 151 patients [13%] [49].

Ulcers: as most patients were reported as having ‘cutaneous ulcerations’ [18,19,41,65] without providing the exact location, we were unable to assess ‘digital ulcers’. 307 patients [42%] had skin ulcers either at the time of inclusion in a study or during the follow-up observation. In 2 retrospective South African studies with greater detail, the frequencies of ever having had any ulceration were 71% (of 174 patients) and 44% (of 151 patients) [5,49].

Renal complications: renal involvement was documented in only 1% of all patients. Among the total of 25 patients in whom kidney function was reported, renal crisis was observed in 9 patients [36%], chronic kidney disease in 2 patients [8%], and proteinuria in 6 patients [24%]. Three retrospective South African studies found renal complications in 5 of 174 [3%] [5], 7 of 58 [12%] [45], and 1 of 52 SSc patients [2%] [86].

Interstitial lung disease (ILD): the definition of ILD was not specified in most publications. When we adjudicated all of radiographic evidence of lung fibrosis, restriction on lung function testing, or chronic cough and dyspnoea, the prevalence of ILD was 51% in 1039 SSc patients with available information on any of these items. Radiographic evidence of lung fibrosis was positive in 237 [44% of 534 patients with ILD]; in 128 [54%] by plain chest X-ray and in 109 [46%] patients by CT-scan. The presence or absence of ILD, if defined only as radiographic evidence of fibrosis, or an FVC below 80% of predicted, was reported in 885 [47% of all patients]. Among those, parenchymal lung fibrosis was observed in 440 [50%] of patients. A study from Kenya reported ILD by imaging or pulmonary function testing in 18 of 50 [36%] of SSc patients [44].

Spirometry results were reported in 184 of all SSc patients [6-8,10,13,14,17,19,24,26,27,29,38,39,41, 45,47-49,85], either as percentage of predicted, or as a dichotomized result (normal/abnormal). 183 of these patients featured a reduced FVC. In 110 patients, the FVC was reported as percentage of predicted; on average these patients showed a FVC of about 70% predicted.

Data on DLCO was sparse. In one South African study, the DLCO was impaired in 86% of 63 SSc patients [45]. More detailed information about the extent of DLCO decline was reported in another South African study that showed an average DLCO of 65% [49]. Pulmonary hypertension (PH): in 576 [31%] patients data on PH was recorded. 159 patients [30%] were deemed to have PH, mainly by echocardiographic pressure estimates [92%]; proof of PH by right heart catheterisation was provided in only 14 patients [8%]. In a Senegalese study on cardiovascular manifestations, PH was diagnosed in 5 of 29 SSc patients [17%] [59]. In Kenya PH was reported in 10 of 50 SSc patients [20%] [44]. In one retrospective South-African study, PH was diagnosed in 7 of 54 patients [13%] [45]. In 6 of these 7 patients, PH was accompanied by lung fibrosis. In a similar study from Dakar, Senegal, 12 out of 83 patients [14.5%] had PH [39]. Four of the 12 Senegalese PH patients had concomitant ILD.

Heart involvement: SSc-specific cardiac manifestations, including a reduced left ventricular ejection fraction, diastolic dysfunction, arrhythmias, pericardial effusion or where no obvious non-SSc cause of heart disease was evident, were reported in a total of 524 [28% of all patients], of whom 144 [28%] had heart involvement. Heart failure was reported in 27 [33%], arrhythmia in 33 [40%], pericarditis/pericardial effusion in 16 [20%] and myocardial infarction/damage in 6 [7%] of patients. In more detailed studies from Senegal, cardiac failure in terms of a depressed left ventricle ejection fraction was observed in 13 of 92 [14%] patients [41] and in a Kenyan study myocardial involvement in 11of 50 [22%] patients [44].

GIT involvement: GIT symptoms were addressed in almost half of all publications. Oesophageal involvement, including peptic lesions of the oesophagus, general oesophageal symptoms and heartburn, were the most frequent complaint and reported in 261 patients [70%] of 373 patients with available information. Heartburn as a symptom of reflux was explicitly reported as present in 113 patients and as absent in 1 patient. The prevalence of dysphagia was 37% (153 patients). Thus, dysphagia appears to represent the second most common GIT manifestation. The highest reported dysphagia prevalence was 66% in a study of 61 patients [45] and the lowest dysphagia prevalence was 8% in a study of 24 patients [48]; both studies were from South Africa.

Musculoskeletal manifestations: ‘arthritis’ was reported in 317 patients [50%] but the definition of arthritis was mostly not provided. Similarly, tendon friction rubs were reported to be present in 15 [0.3% of all SSc patients] patients. Muscle pain was reported to be present in 83 [39% of all SSc patients] and myositis in 58 patients [28]. Creatine kinase elevation was not reported.

Autoantibodies: antinuclear antibody (ANA) findings were reported in 1045 [46% of all patients]. Of these, 674 [65%] were ANA positive. Autoantibody specificities are shown in Table 3. Anti-fibrillarin autoantibodies were the most frequent SSc-specific ANA, followed by anti-topoisomerase 1 autoantibodies. RNA polymerase 3 and anti-centromere autoantibodies were infrequent; in one study of 73 black South African patients none of the patients had anti-centromere antibodies [42].

**Table 3:** autoantibody specificity in 1145 patients with available autoantibody data

Antibody specificity	ANA tested n	ANA positive n	% of antibody tested
ANA positive	1045	674	64.5
**SSc-specific ANA**			
Topoisomerase 1	822	103	12.5
Centromere	382	35	9.2
Fibrillarin (U3-RNP)	85	14	16.5
RNA polymerase 3	84	6	7.1
**Other ANA**			
PM-Scl	84	2	2.4
Jo-1	29	2	6.9
U1-RNP	335	84	25.1
Sm	55	5	9.1
SSA (Ro)	324	72	22.2
SSB (La)	309	30	9.7
dsDNA	122	4	3.3
**Antiphospholipid antibody specificities**			
Antiphospholipid	41	26	63.4
Anticardiolipin	49	3	6.1
Beta-2 glycoprotein 1	64	23	35.9

There was a high prevalence of antiphospholipid antibody specificities (anti-beta-2 glycoprotein-1, anti-phospholipid and anti-cardiolipin). These data were replicated in a Senegalese cross-sectional study that systematically analysed the association of anti-phospholipid antibodies with SSc, the lupus anticoagulant was found in 58% of patients [64]. The persistence of these autoantibodies at repeat testing was however not analysed and a clinical correlation with the antiphospholipid antibody syndrome not made [64].

Mortality: death was reported in 160 [9% of all SSc patients]; in most patients [83%] the cause of death was unknown. Pulmonary complications as the cause of death were reported in seven patients [25%]. Among patients with ILD, the mortality was high; 44.4% died as a consequence of ILD, followed by infection (in 22.2%) [49]. Heart involvement as a cause of death was described in ten patients [36%], and as a result of renal complications in five patients [18%]. Single cases of sudden death, cancer, meningitis, malabsorption, and septicaemia were also reported. In a South-African retrospective series, 20% of patients were known to have died; after a mean follow-up period of 45 months, 63% of deaths were directly attributed to SSc, mostly due to interstitial or vascular lung involvement or renal crisis [93].

Comorbidities: many SSc patients had medical comorbidities. Among the autoimmune diseases, reports mentioned systemic lupus erythematosus overlap in 18 patients [1% of all patients] [10,31,41,44,65] and dermatomyositis in 10 patients [0.5% of all patients] [13,39,41,44]. Further comorbidities were also reported rarely; Sjogren´s Syndrome in 9 patients [0.5% of all SSc patients] [41,44] and rheumatoid arthritis in 4 patients [0.2%] [41,44].

In terms of infections, ten of a total of 154 analysed SSc patients were HIV-seropositive [7%] [17,49]. There was no difference in HIV prevalence among patients with or without ILD [49]. Tuberculosis was diagnosed in 22 patients [13,29,38], of which 17 [77%] had latent tuberculosis [10,48,92] and the remaining 5 patients [23%] had active tuberculosis. Information about TB and HIV comorbidity was however not analysed in the vast majority of patients [98% and 92% respectively]. In one patient, ILD was complicated by concomitant aspergilloma [6].

SSc treatment: treatment mostly consisted of symptomatic medication; patients were often prescribed nonsteroidal anti-inflammatory drugs. Reflux was mostly medicated with proton pump blockers. The oldest study from 1945 reported SSc treatment with parathyroid hormone, vitamins and intravenous calcium [3]. Details on immunosuppressive treatment were provided predominantely in case reports, immunosuppressants were used in up to 80% of patients [44]. Two larger studies from Kenya and South Africa reported glucocorticosteroids as the immunosuppressant most frequently used (50% of patients and 62% respectively) [44,51]. Often relatively high glucocorticosteroid doses were administered (40-60mg per day) [10,53,57]. Methotrexate was used in 5% and 22.5% of patients [44,51]. Immunosuppressants less frequently administered were mycophenolate mofetil, intravenous cyclophosphamide, and azathioprine [44,49]. Three publications reported SSc treatment with hydroxychloroquine [40,44,60], the two case series studies among these reported hydroxychloroquine treatment in 10% [44], and 25% [40] of patients. Treatment with potential antifibrotic agents such as potassium-paraaminobenzoate [25] and D-penicillinamine [44,49,60] was also reported. A South African patient with renal crisis was treated with the angiotensin converting enzyme inhibitor captopril enabling cessation of haemodialysis [32]. Hematopoietic stem cell transplantation was not reported.

## Discussion

This systematic review revealed that in sub-Saharan Africa, publications mostly come from South Africa, Nigeria and Senegal, potentially reflecting the gross domestic product and medical research budget of these nations [99,100]. More than half of the sub-Saharan countries had, however, no information on SSc. Nevertheless, research on SSc increased recently; about half of all reports were published in the last decade. Most patients [83%] were female. The sex ratio of 5.4:1 found in sub-Saharan Africa is well within the range reported in the large multinational database of the European Scleroderma Trial and research (EUSTAR) group (6.7:1), in other continents (3:1) and for African Americans (3.2:1) [2,101,102]. There is no indication that the sex ratio of SSc patients from sub-Saharan Africa differs significantly from that in other geographic regions. In this review, several studies reported a link between SSc and silica exposure [4,48,91,92]. Of our 1884 patients, only in 141 SSc patients a silicate exposure was noted. Regarding the interaction of silica dust and SSc unfortunately no statement can be made as we lack control groups.

More than 70% of patients in sub-Saharan SSc were reported to have diffuse cutaneous SSc. In the United States, diffuse SSc was reported in 60% of African Americans [103]. The high proportion of diffuse SSc in sub-Saharan Africans may in part be explained by economic factors situation and the organization of the health service in which patients with overt and extensive skin involvement are more likely to seek medical attention, while those with more subtle features do not, or are not referred for specialist care. Several papers [12,46,53,54,57] reported skin hypopigmentation frequently providing a ‘salt and pepper’ appearance of the skin. This feature is associated with anti-fibrillarin autoantibodies and seems to be much more than in the USA and Europe [104].

The overall prevalence of RP was 78% in our analysis, but the prevalence varied substantially across different sub-Saharan countries; the larger studies reported prevalences between 57% [41] and 96% [90] which is lower than the prevalence of 96% reported from EUSTAR [105]. Moreover, RP as the initial complaint of SSc occurred in only 16% of sub-Saharan patients as compared to 87% of the mostly Caucasians in EUSTAR [106]. This marked difference in RP prevalence might be due to a number of reasons. Firstly, differences in climatic conditions which modulate this vascular complication, with warmer climates in most sub-Saharan countries compared to European countries, is one explanation. An analysis of the EUSTAR database however did not show a significant relationship between prevalence of RP and ambient temperatures [101]. Studies in the US have not revealed differences in RP prevalence between African-Americans and other racial group [107]. Another possible reason might therefore be that RP symptoms are less likely to trigger a medical visit or are considered worth noting in low income countries. Finally, the difficulty of detecting the blue and red skin colour changes in darkly pigmented individuals may explain in part not only the low RP prevalence, but also the low prevalence of telangiectasia. In a recent South African study of cutaneous lupus, black patients were less likely to have the typical erythematous malar rash compared non-black patients [108].

Anti-topoisomerase 1 autoantibodies were detected in about one quarter of African American SSc patients [2,103]. The prevalence of anti-topoisomerase 1 autoantibodies in our study was somewhat lower, but the prevalence of the other ANA specificities similar with studies of African-Americans in which ANA were present in 87% of patients [103], anti-fibrillarin in 19% [2] and anticentromere in about 10% [103] and 7% [2] respectively.

With regards to the causes of death, about two thirds of all SSc patients appear to die from cardiorespiratory failure secondary to interstitial lung disease, primary or secondary PAH (Mohammed Tikly, personal communication). This systematic review could however not mirror these observations due to lack of detail and follow-up. Further research is clearly needed.

An interesting fact is that SSc in combination with HIV was reported very rarely. The combination with HIV was reported in 7% of patients with known HIV-status. It is unclear if SSc improves during the course of untreated HIV infection similar to the observation in some of the other connective tissue diseases, and if HIV-induced CD4-positive T-lymphocyte loss may account for the low prevalence of some SSc manifestations [109].

This review has systematically analysed considerable patient numbers in heterogeneous study designs. A likely bias result on the one hand from unbalances the various sub-Saharan countries from which reports are available, and differences in health care access in these countries on the other hand. It should also be noted that almost half of the patients were not formally reported to be classified as SSc according to international criteria particularly the recently formulated ACR/EULAR criteria which are more sensitive. Lastly but not least, it must be noted that not all observations can necessarily be attributed to black patients, as exemplified by South Africa, which has a high proportion of other ethnicities.

## Conclusion

SSc is no rarity in sub-Saharan countries but its presentation differs from that in Caucasians. Diffuse skin involvement and focal skin hypopigmentation seem to appear more frequently. Prevalence of anti-fibrillarin autoantibodies seem to be higher likewise. More research must be undertaken to reduce bias and to elucidate the effects of tuberculosis and HIV-infection on SSc presentation and outcome

### What is known about this topic

- The presence of SSc has been described in a variety of sub-Saharan countries;
- Robust data of SSc serology and organ manifestations in sub-Saharan Africa are lacking.

### What this study adds

- Analysis and overview of the published clinical data on systemic sclerosis (SSc) in sub-Saharan Africa.
